# Headache Attributed to Temporomandibular Disorder and Primary Cough Headache

**DOI:** 10.3390/neurolint14010011

**Published:** 2022-01-17

**Authors:** Keita Takizawa, Kentaro Urata, Rena Tanaka, Kana Ozasa, Andrew Young, Noboru Noma

**Affiliations:** 1 Department of Oral Diagnostic Sciences, Nihon University School of Dentistry, Tokyo 101-8310, Japan; takizawa.keita@nihon-u.ac.jp (K.T.); tanaka.rena@nihon-u.ac.jp (R.T.); deka18006@g.nihon-u.ac.jp (K.O.); 2 Department of Complete Denture Prosthodontics, Nihon University School of Dentistry, Tokyo 101-8310, Japan; urata.kenntarou@nihon-u.ac.jp; 3 Department of Diagnostic Sciences, Arthur Dugoni School of Dentistry, University of the Pacific, San Francisco, CA 94103, USA; ayoung@pacific.edu

**Keywords:** temporomandibular disorder, primary cough headache, indomethacin, orofacial pain

## Abstract

Orofacial pain is a frequent chief complaint of many systemic disorders. A primary cough headache may mimic the clinical symptoms of a temporomandibular disorder (TMD) or may be associated with TMDs. Case report: A 52-year-old man presented with a 1-year history of TMD symptoms with clicking. He presented with the chief complaint of a sudden and severe headache when coughing, sneezing, or crouching. Comprehensive intra- and extra-oral examinations were performed, which revealed myofascial pain involving the right masseter and temporalis muscles, disc displacement with reduction in the right temporomandibular joint, and headache attributed to TMD, but no severe headaches appeared in the cough-induced test at the first visit. Initially, we advised the patient to minimize activities that require jaw function (e.g., chewing), avoid jaw parafunction (e.g., bruxism), and to perform at-home jaw exercises to stretch the jaw muscles. The patient’s symptoms reduced by more than half after the TMD home care and physiotherapy. He was then treated with 75 mg of indomethacin per day, which eliminated his headache. The patient was then referred to a headache specialist, who diagnosed primary cough headache.

## 1. Introduction

Primary cough headache (PCH) is coded as 4.1 in the International Classification of Headache Disorders, version 3 (ICHD-Ⅲ). Headaches in category 4 are collectively called “Other primary headache disorders,” with the first four being “Headaches associated with physical exertion” (the other three being 4.2 Primary exercise headache, 4.3 Primary headache associated with sexual activity, and 4.4 Primary thunderclap headache) [1]. The original name for primary cough headache was “Valsalva-maneuver headache,” which was later changed to “benign cough headache.” This was then changed to “primary cough headache” (PCH) in The International Classification of Headache Disorders, version 2 (ICHD-II) [2]. In ICHD-III (2019), new criteria were established for PCH [1].

PCH is a distinct syndrome of headache of sudden onset, which lasts from one second to two hours, peaks almost immediately, and then subsides over several seconds to a few minutes. It is caused by, and occurs in association with, coughing, straining, and/or Valsalva maneuver. Generally, PCH involves the bilateral temporal and posterior head region [3].

Dentists often encounter cases of headache attributed to temporomandibular disorder (TMD) (11.7 in the ICHD-III) [1]. Headache attributed to TMD is diagnosed when all of the following are present: (1) headache in the temporalis muscle, (2) myalgia, myofascial pain, or arthralgia, (3) pain modified by jaw movement, function, or parafunction, and (4) familiar headache in the temporalis muscle from palpation of the temporalis or from jaw range of motion [4]. Dentists usually have the ability to distinguish headache attributed to TMD from migraine and tension-type headaches, as all are quite common [5,6]. In contrast, PCH is a rare condition, which accounts for 1% or fewer of all headache cases encountered in neurologic clinics. Herein, we present a case of PCH accompanied by TMD.

## 2. Case

A 52-year-old man presented with temporomandibular joint (TMJ) pain accompanied by a headache. He had a one-year history of temporomandibular disorder (TMD) symptoms, such as TMJ sounds and TMJ-associated headache, as well as masticatory muscle, neck, and shoulder pain.

Approximately 6 months before presenting at our clinic, the patient had a sudden and severe headache when coughing, sneezing, or crouching. This severe headache occurred about five times a month. He also had a headache while playing tennis. His family reported that he was snoring while sleeping and had bruxism at night.

The patient had two different types of headache: One type was constant, dull, and mild, and had associated neck and shoulder pain. The other type was a moderate-to-severe stabbing pain, developed immediately after coughing, and subsequently changed to a dull pain. This brief headache was localized to the right temporal and parietal regions (sometimes the forehead or supraorbital area) and lasted approximately 10 min (with severe pain for the first 5 min).

The patient had consulted a neurosurgeon. He had undergone magnetic resonance imaging (MRI) to rule out secondary headache; the results were negative for any intracranial disease. He had taken a muscle relaxant (tizanidine hydrochloride) and loxoprofen for 6 months, but these medications did not provide any significant improvement. During the same period, he also had a dull pain around the inside of the eye, and he visited an ophthalmologic clinic to rule out headaches secondary to an ophthalmic condition. The ophthalmologic examination revealed no abnormal findings. 

## 3. Examination

The TMJ examination indicated an active range of motion of 51 mm; disc displacement with reduction was observed in the right TMJ during opening. Muscle palpation elicited severe tenderness in the temporalis muscle on the right side and reproduced the dull headache, but did not reproduce the stabbing headache or the eye pain. The results of the intraoral examination indicated extensive tooth wear but revealed no gingival tenderness. 

**Figure 1:**
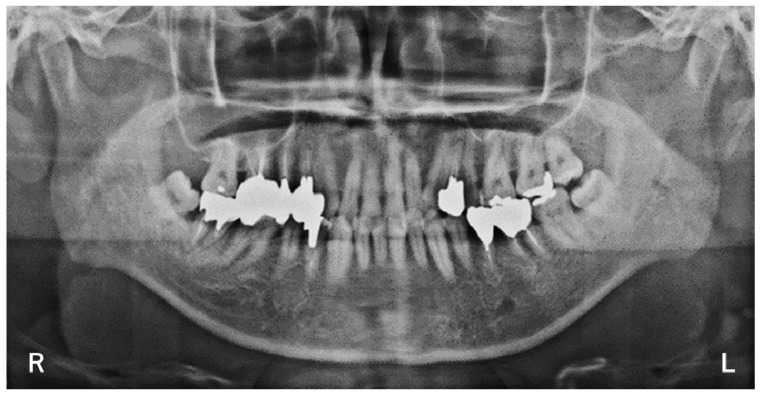
A panoramic radiographic view of the TMJ showed a normal shape in both condyles. R: right. L: left.

A standard radiographic view of the TMJ and panoramic radiographs showed a normal range of motion in both TMJ condyles and no abnormal findings involving the teeth, TMJ, or maxillary sinuses (Figure 1). Cranial nerve screening results were within normal limits.

According to the Diagnostic Criteria for TMD (DC/TMD) [4], the patient’s symptoms were indicative of myofascial pain involving the right masseter and temporalis muscles, disc displacement with reduction in the right TMJ, and headache attributed to TMD.

**Figure 2:**
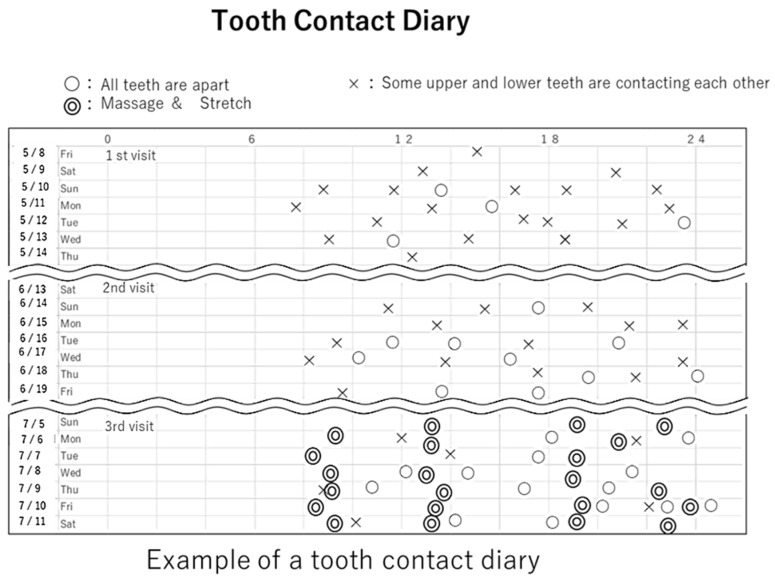
The contact status of the upper and lower teeth.

At his initial visit, the patient was asked to record his tooth contact status in a diary until his second visit (Figure 2). From that diary we calculated the tooth contact rating (TCR), which is TCR = Number of ×/ (Number of × + Number of O) × 100 (%), which is a measure of how frequently teeth are in occlusion (rather than at rest) [5]. The TCR of the patient was high, at approximately 80%. On the second visit, the patient was instructed to avoid daytime jaw parafunction by adopting a mandibular resting position, with upper and lower teeth separated. On the third visit, he was strongly encouraged to perform at-home physical therapy (five sessions a day of temporal and masseter muscle massage at specific tender points, and temporal, masseter, and medial pterygoid muscle stretching). The intensity of the sudden and severe headache decreased from 3 to 2 on the VAS after this regimen. TCR decreased to 20~30%.

We then considered the possibility of PCH, since the signs and symptoms of this patient met the diagnostic criteria for PCH in ICHD-III (Table 1) [1], and prescribed 75 mg of indomethacin (Inteban, Kagawa, Japan) per day, which eliminated the headache. The patient was then referred to a headache specialist, who diagnosed PCH. The patient’s PCH is being monitored by a headache outpatient clinic. The TMD continues to be managed in the orofacial pain clinic.

We asked the patient to wear a reminder (a rubber band on the wrist and an artificial nail) and, on seeing the reminder he was asked to mark an open circle “o” or a “×” in the diary immediately after checking the status of the tooth contact. An open circle meant that the upper and lower teeth were completely apart, and a “×” meant that there was some contact [5].

Differential diagnosis: Migraine. Tension-type headache. Trigeminal autonomic cephalalgia.

## 4. Discussion

To the best of our knowledge, this is the first report of a patient with both PCH and headache attributed to TMD. Clinicians have previously described migraine, cluster headache, chronic paroxysmal hemicrania, and trigeminal neuralgia presenting as toothaches [5,6,7,8]. In one case report, Moncada et al. reported a cough headache presenting as a unilateral toothache [9]. That patient’s pain was located in the right maxillary region and radiated to the ipsilateral temporal region, occipital region, and ear. The patient had undergone two root canal treatments in the upper right first and second bicuspids, followed by tooth extraction. These treatments were not successful in alleviating the pain [9].

Headaches associated with referred pain exhibit secondary central nervous system (CNS) effects that may confuse the clinician. Referred pain may be the cause of the atypical orofacial pain sites in patients with headaches, as the somatic part of the trigeminal nerve (V2 and V3) may converge with the visceral part (V1) of the trigeminal nucleus [10]. The trigeminocervical nucleus is a region of the upper cervical spinal cord where sensory nerve fibers in the descending tract of the trigeminal nerve are thought to interact with sensory fibers from the upper cervical roots. The convergence of upper cervical and trigeminal sensory pathways allows referred pain between the neck and trigeminal sensory receptive fields of the face and head [11]. In this case, we considered that two conditions, namely PCH and headache attributed to TMD, may have developed independently, for two reasons. First, the patient’s TMD developed before the PCH. Second, although 1 kg of palpation pressure was applied for the full 5 s to allow for enough time for spreading or referred pain to manifest itself [4], the patient complained of a dull headache, but did not feel the sharp, severe headache. These observations suggested that PCH and masticatory myofascial pain symptoms were presenting simultaneously, though with different origins [4]. 

Involuntary nonfunctional tooth contact is believed to be an important cause of TMD and headache [12]. Some researchers reported that masticatory myofascial pain symptoms and headache are more prevalent in TCR than in control groups [13,14]. In this case, since the patient’s TCR was high between his first and second visit, we instructed him to avoid daytime jaw parafunction by encouraging a mandibular resting position, with upper and lower teeth separated. After TCR instruction and home jaw exercises to stretch the jaw muscles, the pain intensity and frequency of both headaches were reduced. TMD might have affected the mechanism of the PCH.

In this case, the patient also had neck and shoulder pain. TMD and tension-type headache commonly cause this presentation. Though our patient met the criteria for headache attributed to TMD, and not for tension-type headache, myofascial TMD pain and tension-type headache disorders may overlap, and appear to share many of the same pathophysiological mechanisms [15]. A clinical study demonstrated that alleviation of masticatory muscle tenderness by repeated stretching elevated the pressure pain threshold (PPT), not only in the masticatory muscle but also in the trapezius and brachioradial muscles [12]. Repeated stretching and massage may elevate the threshold of peripheral nociceptors in the affected muscles, and may suppress pain processing and/ or promote pain modulation in the central nervous system [16]. 

The P2X3 receptor plays a role in nociception transmission of orofacial pain in TMD patients. In an animal study, Sun et al. observed that the upregulated expression of P2X3 receptors in the trigeminal subnucleus caudalis (Vc) and midbrain periaqueductal gray (PAG) contributed to the development of hyperalgesia of the masticatory muscles induced by occlusal interference [17]. Expression of P2X3 receptors also was observed in airway afferent nerves and mediated hypersensitivity of the cough reflex. Abdulqawi et al. demonstrated that the systemic P2X3 antagonist dramatically reduced hypersensitivity of the cough reflex and suppressed the cough [18]. One may hypothesize that repeated stretching and massage downregulates expression of P2X3 receptors, resulting in pain regulation in the central nervous system and reducing PCH.

In the present case, loxoprofen was not effective against the headache, but indomethacin (75 mg/day) was. Indomethacin, which may be more effective in general against PCH compared to other non-steroidal anti-inflammatory medications such as loxoprofen, may achieve its effect through a reduction in intracranial pressure via vasoconstriction. According to a previous report, the recommended indomethacin dose for treating PCH is 50–200 mg/day, but a few symptomatic cough headache cases have also been reported to respond to this treatment [1]. Chen et al. reported that the response to indomethacin was higher in PCH than in secondary cough headache [19]. However, another study demonstrated that most patients with primary and secondary cough headaches were responsive to treatment with indomethacin [20,21,22].

The new ICHD-3 diagnostic criteria for PCH includes a headache duration that lasts between 1 s and 2 h [1]. Originally, the ICHD defined the duration of pain as less than 1 min. The duration was changed to “between 1 s and 3 min” in the ICHD-II, and to “between 1 s and 2 h” in the ICHD-IIIbeta [2,23]. Chen et al. reported on the variability in PCH duration: 62.2% of patients had a headache duration of <1 min, 18.9% had a duration of 1–5 min, 8.1% had a duration of 5–30 min, and 10% had a duration of >30 min [24]. Álvarez et al. observed that the duration of secondary cough headache is longer than that of PCH [25]. In this presented case, the intense pain lasted 5 min, followed by a weak pain for 5 min. Shorter duration may be more likely in CPH cases, but it may be difficult to differentiate CPH from secondary cough headache based on headache duration.

## 5. Conclusions

We observed PCH and headache attributed to TMD occurring simultaneously. PCH may have masked the symptoms of the headache attributed to TMD that would cause diagnostic confusion. Orofacial pain and headache specialists should collaborate to further develop diagnostic procedures and management strategies for TMD and PCH. Thus future clinical research is warranted.

## Figures and Tables

**Table 1:** ICHD-3 diagnostic criteria for Primary cough headache.

Primary Cough Headache
Diagnostic criteria:
A: At least two headache episodes fulfilling criteria B–D
B: Brought on by and occurring only in association with coughing, straining and/or other Valsalva maneuver
C: Sudden onset
D: Lasting between 1 s and 2 h
E: Not better accounted for by another ICHD-3 diagnosis.

## Data Availability

Not applicable.
